# Analysis of Bone Scans in Various Tumor Entities Using a Deep-Learning-Based Artificial Neural Network Algorithm—Evaluation of Diagnostic Performance

**DOI:** 10.3390/cancers12092654

**Published:** 2020-09-17

**Authors:** Jan Wuestemann, Sebastian Hupfeld, Dennis Kupitz, Philipp Genseke, Simone Schenke, Maciej Pech, Michael C. Kreissl, Oliver S. Grosser

**Affiliations:** 1 Department of Radiology and Nuclear Medicine, University Hospital Magdeburg, Leipziger Street 44, 39120 Magdeburg, Germany; sebastian.hupfeld@med.ovgu.de (S.H.); dennis.kupitz@med.ovgu.de (D.K.); philipp.genseke@med.ovgu.de (P.G.); simone.schenke@med.ovgu.de (S.S.); maciej.pech@med.ovgu.de (M.P.); michael.kreissl@med.ovgu.de (M.C.K.); oliver.grosser@med.ovgu.de (O.S.G.); 2 Research Campus STIMULATE, Otto-von-Guericke University, 39106 Magdeburg, Germany

**Keywords:** bone scan, bone scan index, bone metastases, deep learning, radiomics

## Abstract

**Simple Summary:**

Standardized reading schemes, the use of indicators derived from medical images and the use of deep learning-based algorithms become very popular in medical imaging. In this retrospective study, we evaluated the performance of an automatic deep-learning-based algorithm for computer-assisted diagnosis in the field of oncological whole-body bone imaging in nuclear medicine. In addition to prostate cancer, representing a tumor entity evaluated thoroughly using the examined methodology (Bone Scan Imaging (BSI) methodology), a modification of the BSI based standard rating scheme facilitate the use of the methodology for other tumor entities (e.g., breast cancer, lung cancer, hepatocellular carcinoma). Diagnostics in clinical routine can benefit from the examined methodology, mainly due to its sensitivity and the high negative predictive value. Non-pathological bone scans may be easily identified. This may lead to a reduced working load in nuclear medicine departments and may result in an improved and more standardized workflow.

**Abstract:**

The bone scan index (BSI), initially introduced for metastatic prostate cancer, quantifies the osseous tumor load from planar bone scans. Following the basic idea of radiomics, this method incorporates specific deep-learning techniques (artificial neural network) in its development to provide automatic calculation, feature extraction, and diagnostic support. As its performance in tumor entities, not including prostate cancer, remains unclear, our aim was to obtain more data about this aspect. The results of BSI evaluation of bone scans from 951 consecutive patients with different tumors were retrospectively compared to clinical reports (bone metastases, yes/no). Statistical analysis included entity-specific receiver operating characteristics to determine optimized BSI cut-off values. In addition to prostate cancer (cut-off = 0.27%, sensitivity (SN) = 87%, specificity (SP) = 99%), the algorithm used provided comparable results for breast cancer (cut-off 0.18%, SN = 83%, SP = 87%) and colorectal cancer (cut-off = 0.10%, SN = 100%, SP = 90%). Worse performance was observed for lung cancer (cut-off = 0.06%, SN = 63%, SP = 70%) and renal cell carcinoma (cut-off = 0.30%, SN = 75%, SP = 84%). The algorithm did not perform satisfactorily in melanoma (SN = 60%). For most entities, a high negative predictive value (NPV ≥ 87.5%, melanoma 80%) was determined, whereas positive predictive value (PPV) was clinically not applicable. Automatically determined BSI showed good sensitivity and specificity in prostate cancer and various other entities. Particularly, the high NPV encourages applying BSI as a tool for computer-aided diagnostic in various tumor entities.

## 1. Background

Whole-body bone imaging with ^99m^Tc-labeled bisphosphonates is an established diagnostic procedure in the staging and follow-up of skeletal metastases from various tumor entities [1,2,3,4,5,6]. Strictly planar whole-body imaging has an intermediate sensitivity in the detection of metastatic bone lesions (sensitivity (SN) = 86.5%) compared to whole-body computed tomography (CT), (SN = 72.9%), magnetic resonance imaging (MRI), (SN = 90.6%), and positron emission tomography (PET), (SN = 89.7%) [7]. Planar bone scan is inferior in terms of specificity (SP = 79.9%) compared to a combination with ancillary single-photon emission computed tomography (SPECT), (SP = 92.8%), PET (SP = 96.8%), MRI (SP = 95.4%), or CT (SP = 94.8%) [7]. SPECT, including additional low-dose CT (SPECT/(CT)), e.g., performed for attenuation correction and anatomical correlation, can further improve the specificity and negative predictive value (NPV) in detecting bone metastases [2,6,8]. Planar bone scan is still the standard of care due to its availability, lower cost, and ability to assess the whole body [9].

The evaluation of bone scans is observer-dependent and shows significant inter-observer variability [10]. Two decades ago, the bone scan index (BSI) was introduced in patients with prostate cancer to quantify the percentage of metastatic skeletal mass from planar bone scans [11,12]. Following the general trend of using radiomics-based evaluation strategies for feature extraction and diagnostics [13,14], the methodology was implemented for automatic estimation of BSI based on deep-learning techniques using an artificial neural network (ANN) [15,16,17]. The ANN features a conventional multilayer architecture consisting of an input layer (45 nodes), one hidden layer (20 nodes), and an output layer, which is used for image segmentation, detection of areas of increased uptake, and classifying these areas as malignant or benign [15]. The ANN, primarily trained and tested for bone scans in patients with metastatic prostate cancer (training database: 1211 scans), was used to diminish interobserver-based effects [15]. BSI was validated as a prognostic indicator, e.g., in high-risk prostate cancer patients [18]. In contrast, due to false-positive ratings of areas of increased uptake, the BSI methodology is limited in the staging of patients with newly diagnosed prostate cancer [19]. BSI was evaluated thoroughly in prostate cancer, and some data on its performance in breast and lung cancer [20,21,22,23,24,25] are also available. Further validations of the methodology, e.g., for further tumor entities, are lacking.

The aim of this study was to validate the performance of BSI, automatically calculated by an ANN trained with bone scans from metastatic prostate cancer patients, in the detection of osseous metastatic disease from bone scans in different tumor entities. Additionally, we examined the potential for improving BSI methodology in clinical application by using entity-specific cut-off values to distinguish between the absence and presence of bone metastases.

## 2. Methods

### 2.1. Patients

**Figure 1:**
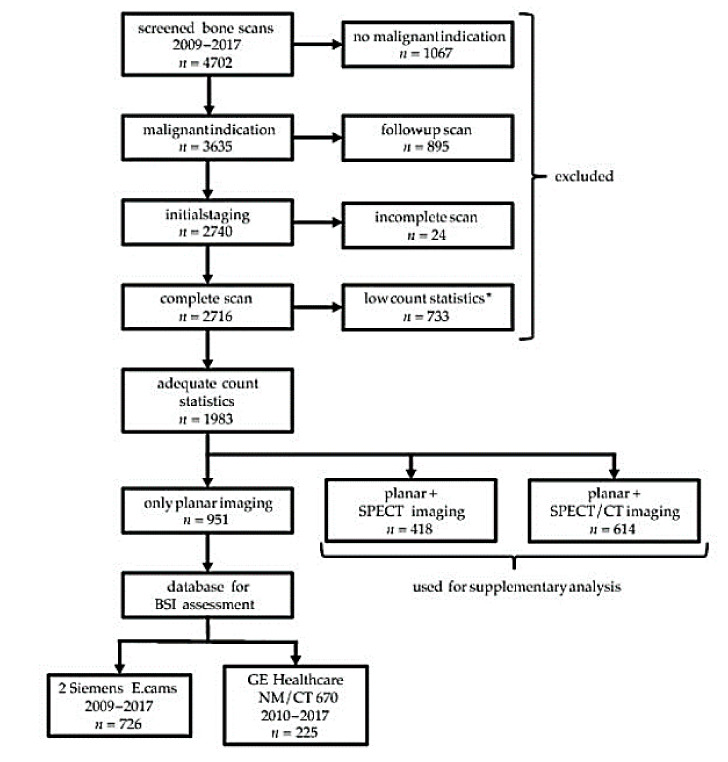
Patient selection for analysis. Note: * excluded from analysis due to an insufficient count statistic (<1 million counts in geometric mean images) to comply with methodological constraints of the BSI algorithm, CT = Computer Tomography, BSI = Bone Scan Index, SPECT = Single Photon Emission Tomography.

The data of 4702 patients referred to our department (Department of Radiology and Nuclear Medicine, University Hospital Magdeburg, Magdeburg, Germany) with a clinical indication for a bone scan between January 2009 and December 2017 were retrospectively screened for analysis. Standardized eligibility criteria were defined as follows to guarantee a comparable data quality for the validation procedure. Patients with suspicion of benign disorders (*n* = 1067), e.g., loosening of joint prosthesis or rheumatic affection, were excluded from further analysis. Only the first whole-body scan of each patient during the observed period was included in the analysis (*n* = 2740). Some data were excluded due to methodological constraints, e.g., an incomplete whole-body scan or signs of extravasation (*n* = 24). Final count statistics had to exceed 1.0 million counts in the geometric mean image, calculated from anterior and posterior projections (*n* = 733 examinations excluded), to ensure the good reliability of the BSI [26]. SPECT/(CT) was performed additionally (*n* = 1032) for localization of suspect tracer accumulation. Corresponding clinical reports presented the condensed information from planar imaging and SPECT/(CT). As a result, for these patients, the classification could have been biased by findings from SPECT/(CT). Patients with SPECT/(CT) data were excluded from primary evaluation because BSI evaluation is based on planar images only (effect from SPECT/(CT); Table S1). Finally, we analyzed a total of 951 bone scans (Figure 1).

The remaining scans were classified according to the primary tumor entity (Table 1). In case of multiple known malignancies (*n* = 11), the current entity triggering the imaging procedure was used for grouping.

All data analyzed were collected as part of routine diagnosis. The retrospective study was conducted in accordance with the Declaration of Helsinki, and the protocol was approved by the local Ethics Committee of the Otto-von-Guericke-University (Medical Faculty and University Hospital Magdeburg), Registration Number: R05/20 (15 July 2020).

### 2.2. Imaging Protocol

Bone scintigraphy was performed by using ^99m^Tc-2,3-dicarboxypropane-1,1-diphosphonate (^99m^Tc-DPD, TECEOS^®^, IBA Molecular, CIS Bio GmbH, Berlin, Germany). The administrated activity was 646 ± 77 MBq. In adipose patients, the injected dose was increased to 11–13 MBq/kg body weight in accordance with the corresponding guidelines [27].

Whole-body planar imaging was performed using one of three scintillation gamma camera systems: (1) two dual-head SPECT gamma cameras of identical design (scanner #1 and #2, model: E.cam, Siemens Medical Solutions Inc., Hoffman Estates, IL, USA) and (2) a dual-head SPECT/(CT) (scanner #3, Discovery NM/CT 670, General Electric, Haifa, Israel). Each camera was equipped with a manufacturer-specific, low-energy, high-resolution (LEHR) collimator. All gamma cameras were monitored by dedicated image quality management procedures. Imaging was performed with a matrix size of 1024 × 256 (pixel size of 2.40 × 2.40 mm (E.cam systems, Siemens Medical Solutions Inc., Hoffman Estates, IL, USA) and 2.21 × 2.21 mm (NM/CT 670)) and a scan speed of 16 cm/min. The energy window was set to 140.5 keV ± 10%. Whole-body imaging was performed using the automatic body contouring system of the gamma camera systems to minimize the detector to patient distance.

In the cases of a two-/three-phase protocol applied for different clinical indications, whole-body images from the late phase (mineralization phase) were used for BSI analysis. Images were acquired in concordance to clinical standard 2.5 to 4 h after injection.

### 2.3. BSI Evaluation

**Figure 2:**
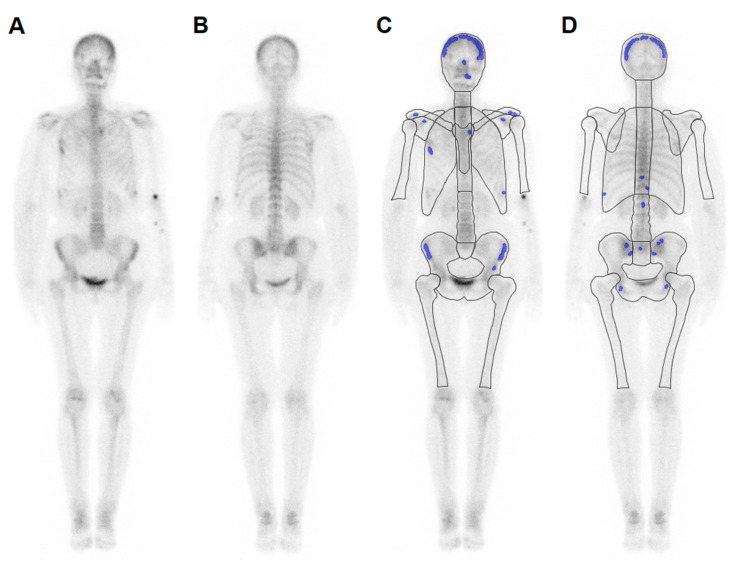
(**A**,**B**) Bone scan of a female patient with breast cancer and (**C**,**D**) results from atlas-based segmentation. Pathologic lesions were automatically segmented (labeled in blue) and correctly rated to be benign (BSI = 0.0%); (**A**,**C**: anterior projection; **B**,**D**: posterior projection).

**Figure 3:**
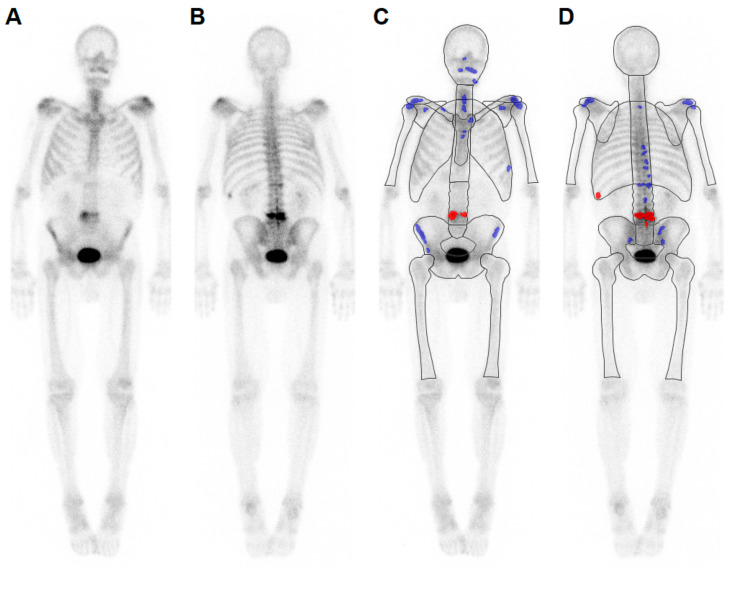
(**A**,**B**) Bone scan of a male patient with colorectal cancer and (**C**,**D**) results from atlas-based segmentation. Three lesions were correctly identified as osseous metastases (lumbar spine and left rib, red labeled). The malignant character is in accordance with the medical report (BSI = 0.54%). Additional benign lesions were labeled in blue; (**A**,**C**: anterior projection; **B**,**D**: posterior projection).

BSI was calculated automatically by dedicated software (EXINI bone, Version 2.1.2, Exini Diagnostics AB, Lund, Sweden). The software automatically segments the anterior and posterior projection of the bone scan by dividing the skeleton into groups (e.g., skull, sternum, cervical spine, thoracic spine, lumbar spine, and pelvis). The bladder was segmented automatically, and counts inside the bladder were excluded from further analysis. Paired bones were distinguished in each planar image into right and left sides (e.g., clavicles, scapulae, ribs, proximal humeri, and femora). Areas of increased tracer accumulation, $A_{i}^{\mathrm{lesions}}$, were highlighted using a region-specific threshold that has to be exceeded in at least 13 contiguous pixels [15]. The threshold values were primarily estimated from training data (bone scans in patients with metastatic prostate cancer) [15]. The integrated ANN calculated the probability of malignancy of the detected areas of increased uptake (Figure 2 and Figure 3). The ratio between projected area of malignant lesions and corresponding area of bone groups, $A_{i}^{\mathrm{bone}},$ were weighted by a factor $w_{I}$ representing the portion of bone in a group regarding total bone mass. This ratio is based on the standard anatomy defined by the International Commission on Radiological Protection [28].

(1) $$\mathrm{BSI}\;=\underset{i}{\sum }w_{i}\times \frac{A_{i}^{\mathrm{lesions}}}{A_{i}^{\mathrm{bone}}}.$$

where BSI represents the total osseous tumor burden (Equation (1)) [11]. Clinical reports defined the reference standard to correlate calculated BSI values regarding absence (M0) and presence (M1) of bone metastases.

### 2.4. Statistics

The R software package (version 3.4.4, The R Foundation for Statistical Computing, Vienna, Austria) was used for all statistical evaluation [29]. Descriptive parameters are expressed as median and range or, in the case of normal distribution, as mean ± standard deviation (SD).

Differences in the BSI values for patients with and without bone metastases were tested for significance by an independent two-samples Wilcoxon’s rank sum test. Receiver operating characteristic (ROC) curves [30] were used to estimate optimal BSI cut-off, distinguishing between bone scans with suspicion of malignancy (detectable metastases) and scans without suspicion of malignancy. Cut-off values were calculated by maximizing the corresponding Youden’s index for each tumor entity. Corresponding sensitivity, specificity, positive predictive value (PPV), negative predictive value (NPV), and area under the curve (AUC) were determined for each type of cancer. For breast and prostate cancer, we performed a sub-analysis to identify a potential effect from the used gamma camera model (E.cam systems vs. NM/CT 670) on performance of the BSI methodology. Differences between ROC curves were analyzed using the DeLong test as a non-parametric approach comparing the corresponding AUCs [31]. The visualization of ROC curves and calculation of AUC values were performed by using the supplementary R software packages ROCR [32] and pROC [33]. Statistical significance was assumed at a *p*-value < 0.05 for two-sided testing.

## 3. Results

### 3.1. Estimation of BSI

**Figure 4:**
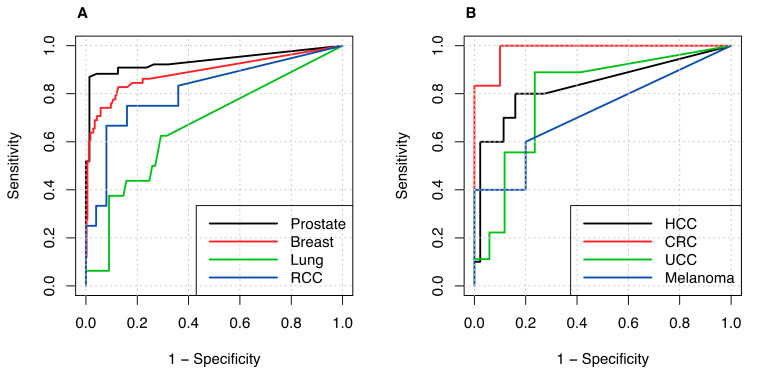
ROC curves: (**A**) tumor entities with osteotropic potential: prostate cancer, breast cancer, lung cancer, and kidney cancer, and (**B**) HCC, CRC, UCC, and melanoma. ROC, receiver operator characteristics; RCC, renal cell carcinoma; HCC, hepatocellular carcinoma; CRC, colorectal cancer; UCC, urothelial cell carcinoma.

For nearly all examined cancer types, BSI was significantly higher in patients with expert-confirmed M1 state compared to the corresponding M0 cohort (Table 1). BSI was highly sensitive (>80%) in detecting metastatic disease (Table 2), with the exception of lung cancer (SN = 62.5%) and melanoma (SN = 60%). Specificity was lower for all tumor entities. ROC curves were generated for all examined cancer types (Figure 4) and BSI cut-offs were calculated (Table 3). The best results in terms of highest AUC were obtained for the BSI in colorectal cancer (AUC = 0.983), prostate cancer (AUC = 0.937), and breast cancer (AUC = 0.890). Hepatocellular carcinoma (HCC) and renal cell carcinoma (RCC) also showed AUC > 0.800, with AUC values of 0.834 and 0.813, respectively.

### 3.2. Effect of Gamma Camera Type on ROC Curve

**Figure 5:**
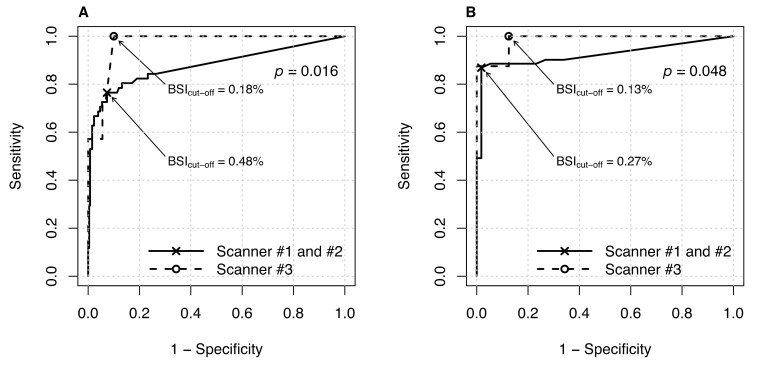
ROC curves for (**A**) breast cancer and (**B**) prostate cancer, illustrating the scanner-specific influence on BSI methodology. The plotted marker (points, stars) reflect the respective best BSI _cut-off_ value for each scanner model.

The data for prostate and breast cancer were used to analyze the impact of the gamma camera model. A scanner-specific ROC analysis was conducted for these specific sub-cohorts. The respective values of the AUC were calculated as 0.923 (scanners #1 and #2) and 0.984 (scanner #3) in patients with prostate cancer, and 0.877 (scanners #1 and #2) and 0.968 (scanner #3) in breast cancer patients. Given these values, a significant effect of the gamma camera model on BSI was detected (Figure 5, prostate cancer, *p* = 0.016; breast cancer, *p* = 0.048). The BSI cut-off estimated for scanners #1 and #2 is higher in both tumor entities compared to the cut-off values estimated for scanner #3 (prostate cancer: 0.27% vs. 0.13%; breast cancer: 0.48% vs. 0.18%).

## 4. Discussion

In the current study, we tested a deep-learning-based BSI methodology for automatic feature extraction and diagnostics from bone scans in various cancer entities. In addition to the standard application in prostate cancer patients, established in clinical routine, we analyzed different tumor entities (e.g., breast cancer and lung cancer) to estimate the test performance using the standard setup of the algorithm. We improved the test performance in detecting metastatic disease by modifying the BSI methodology using entity-specific cut-off values for discriminating M0/M1 states. In parallel, we evaluated the effect of different gamma camera systems on the optimal BSI cut-off value. Data from the two largest sub-cohorts (patients with prostate and breast cancer) were used for this methodological analysis.

The high sensitivity and specificity in detection of bone lesions in patients with prostate cancer, known from the literature, were reproduced [15,19]. Using the standard setup (BSI cut-off = 0), we observed a high sensitivity in patients with prostate cancer, breast cancer, CRC, HCC, RCC, and UCC. This approach was further improved using entity-specific optimized BSI cut-off values (>0%). The specificity in prostate cancer increased (68 to 98.6%) at the cost of a moderate reduction in sensitivity (92 to 87%). Using optimized BSI cut-off values, we observed increased performance in other tumor entities (CRC, HCC, and UCC) up to a certain extent, comparable to results for prostate cancer patients [19,34]. The positive predictive value was partially poor (breast cancer 52%, lung cancer 26%, and HCC 53%), demonstrating that the method is still not readily applicable for the individual confirmation of metastatic disease in these entities. Only in prostate cancer did the relatively low PPV of a BSI > 0 increase (76% to 99%) when applying a cut-off derived from ROC analysis. The BSI showed a high NPV in most examined tumor entities (except melanoma). The NPV remained almost unaffected on a high level after ROC optimization.

A positive BSI in the M0 sub-cohorts is the result of a false-positive classification of benign areas of increased uptake (i.e., degenerative changes, growth metabolism, costal fractures, or inflammation) as malignant even though the algorithm is supposed to reject them. Many scans therefore had a positive definite BSI due to a solitary area of increased uptake, even though there was no obviously suspicious uptake according to the physician’s statement. This effect was already analyzed by Petersen et al. [34], who used BSI cut-off values of 0% and 1% to improve automated M0/M1 differentiation in prostate cancer patients and compare the results to expert opinion. Using a BSI cut-off of 0%, sensitivity (96%) and mean BSI in M0/M1 cohorts were comparable to our results, but specificity (38%) and PPV (21%) were lower. In contrast, applying a BSI cut-off of 1%, which is higher than our optimized BSI cut-off value, increased specificity (98%) at the expense of an unacceptably low sensitivity (58%) for the recognition of metastatic disease. Our ROC-based calculation of entity-specific cut-off values may balance clinical needs.

Sadik et al. [15] reported a comparable sensitivity of 90% and specificity of 89% using a different database of patients with prostate or breast cancer using BSI > 0 as the indicator for metastatic disease. Koizumi et al. [24] examined a different implementation of the examined ANN algorithm using a specific database for Japanese patients. The reported sensitivities were 86%, 82%, and 88% for prostate, breast, and lung cancer patients, respectively. In our study, we roughly reproduced the published results [15,19,24] in terms of sensitivity in prostate (93%) and breast cancer (86%), but we observed a lower sensitivity in lung cancer (69%). The reference data of the tool used by Koizumi et al. [24] (BONENAVI tool, version 2, Fujifilm RI Pharma Co. Ltd., Tokyo, Japan), used for training the specific network, also included bone scans from patients with lung cancer [22,33,35], whereas the software version (EXINI bone V2.1) used here does not. We hypothesized that the higher sensitivity of the alternative software tool is related to the deviating training database with respect to the specific tumor biology of lung cancer (e.g., osteolytic character of metastases). Isoda et al. [21] demonstrated an effect of the tumor entity on the identification of a malignant lesion, i.e., bone metastases from breast and lung cancer had lower computer-rated probability for malignancy compared to prostate cancer. A further explanation, mainly for the poor result in lung cancer, may be the high fraction of lytic lesions, which have a known low malignancy score [21].

The potential bias from the different gamma camera models was assessed by analyzing data from breast and prostate cancer, representing the most frequent cancer entities in our cohort. Comparing ROC curves, we observed significant differences between AUCs and cut-off values. For further examined tumor entities, the evaluation was not applicable due to the limited number of patients in the sub-cohorts. We hypothesize that scanner-individualized (model-specific) training of the ANN and cut-off values can further improve the diagnostic performance of the BSI. A deeper investigation is necessary to substantiate this statement.

The software’s algorithm of the examined tool was trained for a database of bone scans performed in patients (*n* = 1211) with prostate cancer using ^99m^Tc-methylene diphosphonate (^99m^Tc-MDP). In contrast, in our study, imaging was performed with another similar radiopharmaceutical ^99m^Tc-2,3-dicarboxypropane-1,1-diphosphonate. We did not expect this difference to have any significant effect due to comparable pharmacokinetics and accumulation patterns reported by studies conducted during the approval process of the corresponding pharmaceuticals [36,37,38].

A further limitation influencing the performance of the BSI methodology was the kinetics of the tracer. A prolonged time span between administration of the radiopharmaceutical and imaging is known to result in a significantly higher BSI [23,39]. Significant changes in BSI were observed when uptake time was extended from three to four hours post-injection [39]. Since all evaluated bone scans were performed in the context of clinical routine, the uptake time was chosen within the limits of the respective guidelines. No specific optimization of the uptake time was conducted. Established guidelines for clinical routine diagnostics allow considerable flexibility concerning the uptake time (between 2.5 and 4 h post-injection) [27]. Therefore, a variation in the BSI from the wider range of uptake time has to be postulated. An optimized workflow, e.g., optimized uptake time, will improve the methodology. The actual setup represented uncertainty in clinical application.

As ground truth, we used the clinical report from the bone scan in our study. In addition to the individual results from bone scans, the medical report regularly includes further medical information (e.g., from medical history or supplementary imaging). For that reason, an information bias (automated BSI vs. clinical reader with additional clinical information) has to be discussed. The effect was always considered when examining the influence of an additional SPECT or SPECT/(CT) on the clinical decision [9]. This was demonstrated by a sub-analysis based on the cohort with a bone scan and SPECT/(CT) not used for the primary analysis (see Supplementary Material, Table S1). To circumvent this bias, we strictly excluded all bone scans with subsequent SPECT(/CT) from analysis of planar data. The remaining disagreement in information (sole bone scan vs. clinical information, including further imaging, e.g., CT) may also account for discrepancies in rating consistency between the ANN algorithm and the physician. Therefore, the current automatic approach cannot overcome this methodological limitation using a retrospective study setup. Additionally, we hypothesized that further (entity-specific) training of the available ANN for the detection of metastases from other tumor entities would improve BSI performance. Current results should motivate the dedicated training of algorithms with scanner-, collimator-, and tumor-entity-specific databases. The sub-cohorts of our study were not optimized for the specific analysis (e.g., limited by the numbers of cases).

Finally, the BSI is correlated with prognosis and likelihood of response to various therapies in patients with metastatic prostate cancer [40,41,42,43]. The extension of BSI to other tumor entities may open novel opportunities in feature extraction using deep learning technology for observer-independent response prediction and individual prognostication. Although other imaging modalities, in particular PET, were proven to be superior to regular planar bone scans [1,7], lower costs, faster acquisition, and greater availability are reasons why planar bone scan preserves its importance in whole-body skeletal examination. Therefore, this imaging modality still needs to be further improved, e.g., by using automated quantification tools integrating multiparametric data.

## 5. Conclusions

In this retrospective study, we evaluated the performance of an automatic deep-learning-based algorithm for computer-assisted diagnostics in the field of oncological whole-body bone imaging. In addition to prostate carcinoma, representing a tumor entity evaluated thoroughly using the BSI methodology, entity-specific BSI cut-off values facilitate the use of the BSI methodology for other tumor entities (e.g., breast, lung, HCC). Diagnostics in clinical routine can benefit from BSI methodology, mainly due to its sensitivity and the high NPV supporting the identification of non-pathological bone scan.

## Figures and Tables

**Table 1:** BSI values determined in different tumor entities for patients found to have no bone metastases (M0) or having bone metastases (M1) according to clinical report.

Tumor	*n*	Age (Years)	Diagnosis M0		Diagnosis M1		*p*-Value ^†^
			*n*	BSI (%)	*n*	BSI (%)	
	Overall (m/f)	Mean ± SD		Mean (Median; Range)		Mean (Median; Range)	
Breast	406 (12/394)	59.5 ± 13.6	348	0.15 (0.0; 0.0–12.24)	58	4.99 (3.57; 0.0–25.18)	<0.0001
Prostate	149 (149/0)	69.9 ± 7.7	72	0.09 (0.0; 0.0–3.48)	77	8.43 (3.68; 0.0–42.35)	<0.0001
Lung	104 (72/32)	66.5 ± 10.5	88	0.34 (0.0; 0.0–5.56)	16	1.35 (0.08; 0.0–17.35)	<0.0001
HCC	54 (47/7)	67.3 ± 6.6	44	0.17 (0.0; 0.0–5.67)	10	1.40 (0.71; 0.0–6.04)	0.0002 ^#^
RCC	37 (24/13)	64.0 ± 10.5	25	0.37 (0.0; 0.0–6.17)	12	3.24 (0.83; 0.0–6.04)	<0.0001 ^#^
UCC	26 (24/2)	72.3 ± 10.6	17	0.48 (0.0; 0.0–3.93)	9	2.83 (2.04; 0.0–13.90)	<0.0001 ^#^
CRC	16 (13/3)	63.1 ± 12.7	10	0.04 (0.0; 0.0–2.00)	6	2.19 (0.86; 0.10–7.60)	<0.0001 ^#^
Melanoma	15 (11/4)	65.4 ± 9.5	10	0.07 (0.0; 0.0–8.49)	5	2.86 (0.15; 0.0–11.90)	0.13
Other *	143 (81/62)			not analyzed			

M0 = no metastatic involvement of the skeleton according to clinical report, M1 = presence of bone metastasis according to clinical report, BSI = bone scan index, m = male patients, f = female patients, UCC = urothelial cell carcinoma, HCC = hepatocellular carcinoma, RCC = renal cell carcinoma, CRC = colorectal cancer, SD = standard deviation. ^†^ Wilcoxon’s rank sum test (Mann–Whitney–Wilcoxon test). * Not analyzed due to high heterogeneity: this category includes thyroid cancer (*n* = 16), cholangiocellular carcinoma (*n* = 13), gastric cancer (*n* = 11), esophageal cancer (*n* = 8), pancreatic cancer (*n* = 5), neuroendocrine tumors (*n* = 7), head/neck cancer (*n* = 16), sarcoma (*n* = 14), cancer of unknown primary (*n* = 9), gynecologic tumors apart from breast cancer (*n* = 4), testicular cancer (*n* = 4), blood or lymphatic cancer (*n* = 8), mesothelioma (*n* = 1), urachal cancer (*n* = 1), or clinical constellation suggesting a malignant disorder (pathologic fractures, bone pain, hypercalcemia, etc., *n* = 26). ^#^ post-hoc empirical power analysis: HCC 0.71, RCC 0.64, UCC 0.62, CRC 0.67.

**Table 2:** Performance of the BSI as a diagnostic test for bone metastases. Descriptive statistical parameters refer to the decision-making when zero/non-zero BSI is used to distinguish between M0 and M1. A BSI larger than zero indicates osseous involvement according to its actual definition.

Tumor	SN	SP	PPV	NPV
Breast	86.2%	75.3%	36.8%	97.0%
Prostate	92.2%	68.1%	75.5%	89.1%
Lung	62.5%	68.2%	26.3%	90.9%
HCC	80.0%	72.7%	40.0%	94.1%
RCC	83.3%	64.0%	52.6%	88.9%
UCC	88.9%	58.8%	53.3%	90.9%
CRC	100%	70.0%	66.7%	100%
Melanoma	60.0%	80.0%	60.0%	80.0%

SN, sensitivity; SP, specificity; PPV, positive predictive value; NPV, negative predictive value.

**Table 3:** BSI thresholds estimated from receiver operating characteristic (ROC) analysis for each examined tumor entity to distinguish between M0 and M1 situations.

Tumor	*n*	AUC	BSI Cut-Off	SN	SP	PPV	NPV
Breast	406	0.890	0.18%	82.8%	87.4%	52.2%	96.8%
Prostate	149	0.937	0.27%	87.0%	98.6%	98.5%	87.7%
Lung	104	0.663	0.06%	62.5%	70.5%	27.8%	91.2%
HCC	54	0.834	0.13%	80.0%	84.1%	53.3%	94.9%
RCC	37	0.813	0.30%	75.0%	84.0%	69.2%	87.5%
UCC	26	0.797	0.39%	88.9%	76.5%	66.7%	92.9%
CRC	16	0.983	0.10%	100%	90.0%	85.7%	100%
Melanoma	15	0.720	0.15%	60.0%	80.0%	60.0%	80.0%

AUC, area under the curve.

## Supplementary Materials

The following are available online at https://www.mdpi.com/2072-6694/12/9/2654/s1, Table S1: Effect of the information bias from physician’s report (e.g., from added SPECT/(CT)) on performance of BSI for different tumor entities.

## Abbreviations

ANN	artificial neural network
AUC	area under the curve
BSI	bone scan index
CRC	colorectal cancer
CT	computed tomography
DPD	2,3-dicarboxypropane-1,1-diphosphonate
HCC	hepatocellular carcinoma
keV	kiloelectron volts
MBq	Megabecquerel
MDP	methylene diphosphonate, medronic acid
NPV	negative predictive value
PET	positron emission tomography
PPV	positive predictive value
RCC	renal cell carcinoma
ROC	receiver operating characteristic
SN	sensitivity
SP	specificity
SPECT	single photon emission computed tomography
UCC	urothelial cell carcinoma
