# Supplementary material for: Analysis of Bone Scans in Various Tumor Entities Using a Deep-Learning-Based Artificial Neural Network Algorithm—Evaluation of Diagnostic Performance
Source: Cancers (Basel). 2020 Sep 17;12(9):2654. doi: 10.3390/cancers12092654 (PMC7565494; doi:10.3390/cancers12092654)
Supplement: Supplementary file 1 [file cancers-12-02654-s001.pdf]

Supplementary Materials: Analysis of Bone Scans in Various Tumor Entities Using a Deep-Learning-Based Artificial Neural Network Algorithm—Evaluation of Diagnostic Performance

Jan Wuestemann, Sebastian Hupfeld, Dennis Kupitz, Philipp Genseke, Simone Schenke, Maciej Pech, Michael C. Kreissl and Oliver S. Grosser

Table S1. Effect of the information bias from physician's report (e.g., from added SPECT(/CT)) on performance of BSI for different tumor entities.

Tumor	Bone scan		Bone Scan + SPECT(/CT)		*p*-Value †
	n	AUC	*n*	AUC	
Breast	406	0.890	297	0.739	<0.0001
Prostate	149	0.937	164	0.937	0.002
Lung	104	0.663	120	0.678	0.868
HCC	54	0.834	46	0.619	0.086
RCC	37	0.813	46	0.734	0.460
UCC	26	0.797	43	0.808	0.928
CRC	16	0.983	21	0.982	0.963
Melanoma	15	0.720	21	0.731	0.954

AUC, area under the curve. † DeLong test [1].

The BSI was calculated for each subcohort (bone scan vs. bone scan + SPECT(/CT)). BSI algorithm provided higher performance in subcohort with exclusive planar imaging. In the cohort with clinically indicated ancillary SPECT/(CT) the BSI methodology demonstrated lower performance in prediction. Differences in AUC between both cohorts, demonstrating the effect by the information bias (BSI from bone scan vs. physician's report respecting SPECT/(CT)), were evaluated for significance.

References

1. DeLong, E.R.; DeLong, D.M.; Clarke-Pearson, D.L. Comparing areas under two or more correlated reciver operating characteristics curves: a nonparametric approach. *Biometrics* **1988**, *44*, 837–845.

© 2020 by the authors. Licensee MDPI, Basel, Switzerland. This article is an open access article distributed under the terms and conditions of the Creative Commons Attribution (CC BY) license (http://creativecommons.org/licenses/by/4.0/).
